# The liver-brain axis under the influence of chronic Opisthorchis felineus infection combined with prolonged alcoholization in mice

**DOI:** 10.18699/vjgb-25-11

**Published:** 2025-02

**Authors:** D.F. Avgustinovich, I.V. Chadaeva, A.V. Kizimenko, A.V. Kovner, D.V. Bazovkina, D.V. Ponomarev, V.I. Evseenko, V.A. Naprimerov, M.N. Lvova

**Affiliations:** Institute of Cytology and Genetics of the Siberian Branch of the Russian Academy of Sciences, Novosibirsk, Russia Institute of Solid State Chemistry and Mechanochemistry of the Siberian Branch of the Russian Academy of Sciences, Novosibirsk, Russia; Institute of Cytology and Genetics of the Siberian Branch of the Russian Academy of Sciences, Novosibirsk, Russia; Institute of Cytology and Genetics of the Siberian Branch of the Russian Academy of Sciences, Novosibirsk, Russia; Institute of Cytology and Genetics of the Siberian Branch of the Russian Academy of Sciences, Novosibirsk, Russia; Institute of Cytology and Genetics of the Siberian Branch of the Russian Academy of Sciences, Novosibirsk, Russia; Institute of Cytology and Genetics of the Siberian Branch of the Russian Academy of Sciences, Novosibirsk, Russia; Institute of Solid State Chemistry and Mechanochemistry of the Siberian Branch of the Russian Academy of Sciences, Novosibirsk, Russia; Institute of Cytology and Genetics of the Siberian Branch of the Russian Academy of Sciences, Novosibirsk, Russia Novosibirsk State Agrarian University, Novosibirsk, Russia; Institute of Cytology and Genetics of the Siberian Branch of the Russian Academy of Sciences, Novosibirsk, Russia

**Keywords:** mice, Opisthorchis felineus infection, chronic ethanol consumption, liver, brain, microglia, proinflammatory cytokine, behavior, мыши, инфекция Opisthorchis felineus, хроническое потребление этанола, печень, мозг, микроглия, провоспалительные цитокины, поведение

## Abstract

Our purpose was to model a combination of a prolonged consumption of ethanol with Opisthorchis felineus infection in mice. Four groups of C57BL/6 mice were compiled: OF, mice infected with O. felineus for 6 months; Eth, mice consuming 20 % ethanol; Eth+OF, mice subjected to both adverse factors; and CON, control mice not exposed to these factors. In the experimental mice, especially in Eth+OF, each treatment caused well-pronounced periductal and cholangiofibrosis, proliferation of bile ducts, and enlargement of areas of inflammatory infiltration in the liver parenchyma. Simultaneously with liver disintegration, the infectious factor caused – in the frontal cerebral cortex – the growth of pericellular edema (OF mice), which was attenuated by the administration of ethanol (Eth+OF mice). Changes in the levels of some proteins (Iba1, IL-1β, IL-6, and TNF) and in mRNA expression of genes Aif1, Il1b, Il6, and Tnf were found in the hippocampus and especially in the frontal cortex, implying region-specific neuroinflammation. Behavioral testing of mice showed that ethanol consumption influenced the behavior of Eth and Eth+OF mice in the forced swimming test and their startle reflex. In the open field test, more pronounced changes were observed in OF mice. In mice of all three experimental groups, especially in OF mice, a disturbance in the sense of smell was detected (fresh peppermint leaves). The results may reflect an abnormality of regulatory mechanisms of the central nervous system as a consequence of systemic inflammation under the combined action of prolonged alcohol consumption and helminth infection.

## Abstract (ru)

Описторхоз – паразитарное заболевание, вызываемое трематодами семейства Opisthorchiidae, к которому относится вид Opisthorchis felineus, максимально представленный у жителей Обь-Иртышского речного бассейна Западной Сибири. Хроническое употребление алкоголя влечет за собой неизлечимые заболевания печени, которые могут быть усугублены привнесением гельминтной инфекции. Хроническое воспаление на фоне двух воздействий может вызывать нарушения в других органах и системах, в том числе в центральной нервной системе. Сочетание длительного потребления 20 % этанола и инфекции O. felineus моделировали у мышей C57BL/6 с целью исследования изменений в мозге таких животных. Были сформированы четыре группы мышей: OF – мыши, инфицированные O. felineus в течение 6 мес; Eth – мыши, потребляющие 20 % этанол; Eth+OF – мыши с сочетанным действием двух факторов; CON – контрольные мыши, не испытывающие эти воздействия. Оба фактора вызывали у мышей, особенно в группе Eth+OF, выраженные перидуктальный фиброз и холангиофиброз, пролиферацию желчных протоков и увеличение участков воспалительной инфильтрации в паренхиме печени. Одновременно с нарушениями в печени, во фронтальной коре мозга инфекционный фактор обусловливал усиление перицеллюлярного отека (мыши OF), сдерживаемого введением этанола (мыши Eth+OF). Также обнаруже- ны колебания уровней белка (Iba1, IL-1β, IL-6, TNF) и экспрессии генов Aif1, Il1b, Il6 и Tnf в гиппокампе и особенно во фронтальной коре, предполагающие регион-специфическое нейровоспаление. Поведенческое тестирование мышей показало, что потребление этанола влияло на поведение мышей Eth and Eth+OF в тесте принудительного плавания и на Startle-рефлекс. В тесте открытого поля более выраженные изменения происходили у мышей OF. У животных всех трех экспериментальных групп, но особенно у OF, выявлена аномалия обоняния (на свежие листья мяты перечной). Полученные данные могут отражать нарушение регуляторных механизмов в головном мозге вследствие системного воспаления при сочетанном действии длительного употребления алкоголя и гельминтной инфекции.

## Introduction

The family Opisthorchiidae includes three species of trematodes: Clonorchis sinensis, most common in China, Korea, and the Far East of the Russian Federation (35 million people); Opisthorchis viverrini, widespread in Thailand and Laos (10 million people); and Opisthorchis felineus, manifesting the highest prevalence of infection among fish in water bodies of the Ob-Irtysh basin in Russia (1.2 million people) (Mordvinov, Furman, 2010; Petney et al., 2013; Saijuntha et al., 2021). The last two species cause opisthorchiasis when fish of the family Cyprinidae are eaten raw or undercooked. During prolonged helminth infection, mature worms parasitize not only bile ducts of the liver and the gall bladder of piscivorous mammals but also pancreatic ducts, as shown for O. felineus and C. sinensis (Bernstein et al., 1994; Mordvinov, Furman, 2010; Lvova et al., 2023), thereby causing complications such as various forms of pancreatitis (Gundamaraju, Vemuri, 2014). Given that opisthorchiasis upregulates proinflammatory cytokines and leukocytes in the blood (Sripa et al., 2012; Avgustinovich et al., 2022a), this disease is considered a systemic illness that provokes pathologies in other organs and systems of the body (Boonpucknavig et al., 1992; Akhmedov, Kritevich, 2009; Bychkov et al., 2011), including the central nervous system (CNS) (Lvova et al., 2020; Avgustinovich et al., 2016, 2022b). One can expect the development of neuroinflammation during opisthorchiasis, judging by tenets of the “liver–brain axis” paradigm (D’Mello, Swain, 2011). Ac-cording to those authors, three proinflammatory cytokines – interleukin 1 beta (IL-1β), interleukin 6 (IL-6), and tumor necrosis factor (TNF) – in the blood are key promoters of central neural changes in chronic liver diseases. In addition, in the context of liver inflammation, activation of microglia takes place with subsequent recruitment of blood monocytes into the brain. All this strongly drives hepatic inflammationassociated sickness behavior.

The daily alcohol (ethanol) abuse/misuse is a major cause of inevitable damage to the liver (Collins, Neafsey, 2012). The progression of alcoholic liver disease induces cirrhosis, liver cancer, and acute and chronic liver failure and can be fatal (Stickel et al., 2017). As determined by the World Health Organization (WHO. Alcohol. https://www.who.int/news-room/ fact-sheets/detail/alcohol), worldwide, 3 million deaths every year result from overconsumption of alcohol, and this figure represents 5.3 % of all deaths.

As with opisthorchiasis, excessive alcohol consumption entails pathological changes in other organs and systems of organs, for example, in the gut microbiota (Saltykova et al., 2016; Bishehsari et al., 2017; Bajaj, 2019; Ketpueak et al., 2020; Pakharukova et al., 2023; Yao et al., 2023). Aside from the direct negative effect on the intestines, alcohol disrupts bile acid synthesis in the liver during inflammation and impairs bile acid entry into the gallbladder for subsequent secretion into the small intestine. In this context, the reabsorption of bile by the liver is impeded, which under normal conditions is 95 %. Accordingly, in such patients, in addition to alcoholic liver diseases, microbial composition and functions of the intestine change, leading to functional alterations in the “gut–liver–brain axis” (Bishehsari et al., 2017). As a consequence, symptoms of hepatic encephalopathy are aggravated, which are associated with microglial activation and subsequent cognitive deficits (Bajaj, 2019).

On the other hand, alcohol can also have a direct impact on the CNS by inducing cerebral cortical edema and electrolyte (Na+ or K+) accumulation (Collins et al., 1998), and during chronic consumption, alcohol can cause neuronal loss in some brain structures (the cerebral cortex, hypothalamus, hippocampus, and cerebellum) (Harper, 1998; de la Monte, Kril, 2014; Fowler et al., 2014). These effects are attributable to activation of resident microglia and peripheral-macrophage infiltration of the CNS, particularly in the hippocampus, and the two processes together contribute to overexpression of proinflammatory markers in various regions of the brain, including the cortex and hippocampus (Yang et al., 2014; Henriques et al., 2018; Lowe et al., 2020). These phenomena negatively affect cognitive abilities, learning, and memory (Geil et al., 2014).

In humans, these two adverse factors (alcohol consumption and O. felineus infection) can often occur simultaneously. Unfortunately, a combination of ethanol consumption with chronic O. felineus infection can have irreversible consequences for humans, as previously shown in a model of such a combination (Avgustinovich et al., 2022a). In that work, a more pronounced liver pathology was accompanied by splenomegaly due to structural changes in the spleen as well as elevated levels of IL-6 and higher leukocyte and monocyte counts in the blood. Taken together, they meant substantial whole-body inflammation. Considering these data as well as a known statement about possible brain disturbances during severe hepatic inflammation (D’Mello, Swain, 2011), the purpose of our current study was to investigate – by histological, immunohistochemical, and molecular methods – changes in inflammatory markers in the cerebral cortex and hippocampus, as assessed by determination of microglial activation and of expression of proinflammatory cytokines IL-1β, IL-6, and TNF. Because any disturbances in the brain manifest themselves in behavior in mammals, behavioral patterns of mice were assessed here by the open field test (Ramos, Mormède, 1998) and forced swimming test (Porsolt et al., 1977), along with estimation of the startle reflex in response to acoustic signals (Paylor, Crawley, 1997).

## Materials and methods

Animals. Male mice of inbred strain C57BL/6 were obtained from the multi-access center Vivarium of Conventional Animals of the Institute of Cytology and Genetics, Siberian Branch of the Russian Academy of Sciences (ICG SB RAS). All animals were maintained in standard cages (36 × 22 × 12 cm) at 2–5 individuals per cage, on a 12/12 h light/dark cycle, at 24 ± 1 °С with free access to pelleted feed and liquid. The study was conducted in accordance with Directives of the Council of the European Union (86/609/EEC) of November 24, 1986 and according to a decision of the Bioethics Commission of the ICG SB RAS (protocol No. 113 of December 9, 2021).

Obtaining of O. felineus metacercariae. Metacercariae of O. felineus were isolated from naturally infected ides caught in the Ob River (Novosibirsk Oblast) by a method described previously (Avgustinovich et al., 2016, 2021). Metacercariae of O. felineus were administered to mice intragastrically (100 larvae per mouse) using specialized probes (Braintree Scientific, Inc.).

**Fig. 1.**
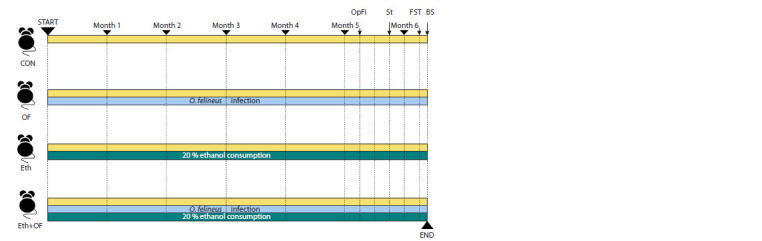
Design of the experiment.

The design of the experiment. As a result, four groups of mice were set up: CON (n = 15), not subjected to any pathogenic procedures; OF (n = 15), infected with O. felineus (duration of infection: 6 months); Eth (n = 15), consuming 20 % ethanol for 6 months; and Eth+OF (n = 13), subjected to both procedures (Fig. 1). Mice were trained to consume ethanol according to a protocol described before (Avgustinovich et al., 2022a). Five months after the infection initiation, the behavior of all mice was recorded in the open field test. By the end of 6 months, the startle response of mice to an acoustic signal (startle reflex) was evaluated. The animals were then housed individually in 28 × 14 × 10 cm cages to evaluate behavior in the forced swimming test. At the end of the experiment, the animals were killed by decapitation, and brain samples were collected for subsequent analyses. The hippocampus and frontal cortex were isolated on ice, placed in liquid nitrogen and then in a freezer at –70 °C until the expression of genes of interest was assayed by real-time polymerase chain reaction (qPCR). Brains from five animals in each group were put in 10 % formalin for subsequent histological and immunohistochemical examination. The liver of these animals was also placed in formalin for subsequent determination of pathomorphological structural alterations in this organ under the influence of the two tested adverse factors.

The open field test. This is one of the most popular tests in behavioral studies, which assesses effects of external factors on rodents (Ramos, Mormède, 1998). When animals are first exposed to an unfamiliar open space, behavioral responses are believed to be mediated by anxiety, whereas repeated exposure can lead to exploration of new objects (or odors) by the animals in a nonthreatening familiar environment (Belzung, 1992; Choleris et al., 2001). For testing, a 40 × 40 cm field made of white plastic material was utilized, which was visibly subdivided into squares (4 × 4) with a side of 10 cm. The illumination at the field level was 320 lx. In the middle of one side of the field, an inverted office metallic tumbler for pencils (hereafter: tumbler) was placed. Five minutes before the testing, the animals were brought into the test room for activation, after which they were always placed in the middle of the wall opposite the wall with the tumbler. Each animal’s behavior was recorded twice (for 3 min each time) using a video camera in the session with an empty tumbler and next with a tumbler containing fresh peppermint leaves. Further analysis of animal behavior was carried out in the Behavioral Observation Research Interactive Software (BORIS) (Friard, Gamba, 2016), in which we determined the number, time (duration), and latency of approaches and/or turns to the tumbler, the number of square crossings, and the number, time (duration), and latency of rearings near a wall.

Startle reflex testing. The startle reflex of humans and animals toward a sharp sound signal is an innate reflex and characterizes the CNS’s ability to filter sensory information (Paylor, Crawley, 1997). The behavioral response of mice to an acoustic stimulus was measured using an SR-Pilot device (San-Diego Instruments, USA). Background white noise was set to 65 dB. Each animal was placed in the chamber, and after 3-min adaptation, 10 impulses (P) of 115 dB intensity and 40 ms duration were presented, alternating with 10 impulses preceded by weak (85 dB, 40 ms) prepulses (PP), which were presented 100 ms before the main impulse. The interval between stand-alone Р impulses and the PP combination was 15 s. The size of the response of a mouse to the stimuli (amplitude) was displayed by the device’s screen in relative units. Prepulse inhibition (PPI, %) was calculated (Paylor, Crawley, 1997) by means of the formula: 100 – [(PP/P) × 100].

The forced swim test. This test is the most popular in research on depressive-like behavior in rodents because it is sensitive to the action of antidepressants (Porsolt et al., 1977). During the testing, transparent plastic cylinders 30 cm high and 9.5 cm in diameter were used, which were filled with water (t = 25 ± 1 °C) to a level of 19 cm. Five minutes before the test, the mice were sequentially brought into the test room and then placed for 5 min into the cylinder filled with water; their behavior was recorded on a video camera. Further analysis was performed in the BORIS software (Friard, Gamba, 2016). The duration of active and passive swimming was assessed; the latter included drift (when the mouse made weak movements with one or two hind legs to keep the head above water) and a state of complete immobility.

RNA isolation for qPCR. To obtain RNA from mouse brain structures, the TRI reagent (Sigma-Aldrich, USA) was employed according to the protocol for samples with a high fat content, then the samples were purified on magnetic particles (Agencourt RNAClean XP Kit, Beckman). The concentration of the resulting RNA was determined using a Qubit™ 2.0 fluorimeter (Invitrogen/Life Technologies) with a kit (RNA High Sensitivity, Invitrogen). Next, the isolated RNA was treated with DNase using the DNase I, RNase-free kit (Thermo Fisher Scientific, USA). Complementary DNA (cDNA) was synthesized by means of a reverse-transcription kit (#OT-1, Syntol, Russia). All stages of RNA isolation and analysis and those of cDNA preparation were carried out according to the protocols of the manufacturers of the corresponding kits

Using the PrimerBLAST web service, we designed oligonucleotide primers for qPCR. qPCR was conducted with the EVA Green I kit (#R-441, Syntol, Russia) according to the manufacturer’s instructions, and amplification efficiency for each primer pair was 90–110 %. qPCR was carried out in three technical replicates on a CFX-96 thermal cycler (Bio- Rad, USA). qPCR efficiency was determined by means of serial dilutions of cDNA (standards); after completion of qPCR, melting curves of the products were constructed to monitor the specificity of the reaction. Expression levels of each analyzed gene were normalized to two reference genes, the expression stability of which was checked in both brain regions under study and in each of the four groups of mice. Based on literature data (Stephens et al., 2011), three reference genes were chosen: Actb (beta actin: a highly conserved protein that participates in cell motility, structure, and integrity), B2m (beta-2-microglobulin: a light-chain component of major histocompatibility complex class I), and Hprt1 (hypoxanthine phosphoribosyltransferase 1: a eukaryotic enzyme of purine metabolism).

The genes of interest were selected taking into account their functional characteristics listed in the Human Protein Atlas (https://www.proteinatlas.org/). The following genes were investigated in the experiment: Aif1 (a marker of microglial activity) and Il1b, Il6, and Tnf (markers of inflammation).

Sequences of the chosen primers were as follows: 1 Actb (F) 5′-TATTGGCAACGAGCGGTTCC (R) 5′-TGGCATAGAGGTCTTTACGG 2 Aif1 (F) 5′-GGATTTGCAGGGAGGAAAA (R) 5′-TGGGATCATCGAGGAATTG 3 B2m (F) 5′-CTGCTACGTAACACAGTTCCACCC (R) 5′-CATGATGCTTGATCACATGTCTCG 4 Hprt1 (F) 5′-GAGGAGTCCTGTTGATGTTGCCAG (R) 5′-GGCTGGCCTATAGGCTCATAGTGC 5 Il1b (F) 5′-ACACTCCTTAGTCCTCGGCCA (R) 5′-CCATCAGAGGCAAGGAGGAA 6 Il6 (F) 5′-ACAAAGCCAGAGTCCTTCAGAG (R) 5′-ACGCACTAGGTTTGCCGAG 7 Tnf (F) 5′-AGCCGATGGGTTGTACCTTG (R) 5′-GGTTGACTTTCTCCTGGTATGAGA

**Fig. 2.**
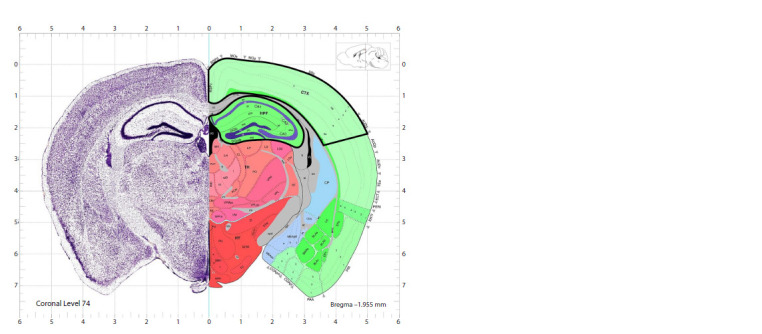
Areas of the cerebral cortex and hippocampal formation (highlighted with a black line on the right) from the brain of C57BL/6 mice, after immunohistochemical analysis The image of the frontal brain section at levels 72–74 was borrowed from the Allen Mouse Brain Atlas (Allen Institute Publications for Brain Science; http://mouse.brain-map.org/static/atlas).

Histological examination of sections of the cerebral cortex and liver. After 10 days of fixation in a 10 % formaldehyde solution, the brain and liver of mice were sectioned for subsequent processing in an STP 120 carousel-type apparatus for automatic incubation in a graded series of ethanol and xylene (Thermo Fisher Scientific, USA). The frontal section separated the middle part of the brain, containing the hippocampus, at level 60–64 according to the Allen Mouse Brain Atlas (http:// mouse.brain-map.org/static/atlas). Samples containing bile ducts and parenchyma and measuring 100 × 150 × 70 mm were dissected from the large lobe of the liver. After dehydration, the tissue samples were embedded in the HISTOMIX synthetic paraffin medium (Russia) by means of an EC-350 embedding station (Thermo Scientific, USA). Sections with a thickness of 3.5‒4.0 μm were prepared on a Microm HM 355S rotary microtome (Thermo Fisher Scientific, USA). To study the cortex and hippocampus, brain samples were cut to levels 72–74 according to the brain atlas (Fig. 2). The obtained samples of the cortex and liver were subsequently stained by standard techniques: basic survey staining with hematoxylin and eosin and the Masson method (staining of connective tissue). After that, sections mounted in BioMount (BIO-OPTICA, Italy) were visualized under an Axioskop 2 plus microscope equipped with an AxioCam MRc camera (Carl Zeiss, Germany) and AxioVision software (release 4.12). Morphometry of structural changes in the cerebral cortex was performed using a closed test system targeting 100 points with an area of 3.64 × 105 μm2. Meanwhile, numerical density of perivascular and pericellular edema and the total number of blood vessels were assessed. The scoring method for these measurement data had been described in detail earlier (Pakharukova et al., 2019). In the liver, the absence/presence of periductal fibrosis, cholangiofibrosis, and inflammatory infiltration and proliferation of bile ducts was recorded

Immunohistochemical analysis of sections of the hippocampus and cerebral cortex. The hippocampus and cortex at levels 72–74 of frontal brain sections were examined on glass slides with an adhesive poly-L-lysine coating (BVS, Russia). To count activated brain microglia cells and cells synthesizing proinflammatory cytokines, we utilized an indirect biotinfree peroxidase immunohistochemical technique for staining paraffin sections using a kit (SpringBioScience Kit HRP-125, Pleasanton, CA, USA) and primary antibodies specific to ionized calcium-binding adapter protein 1 (Iba1) (Abcam, cat. # ab5076, dilution 1:300), IL-6 (Abcam, ab6672, 1:100), TNF (Abcam, ab6671, 1:100), and IL-1β (Abcam, ab9722, 1:100). According to this technique, after standard deparaffinization and dehydration, antigens were retrieved on the sections in citrate buffer (pH 6.0) in a microwave oven at 700 W for 5 min. After washing four times in phosphate-buffered saline (PBS, pH 7.6), endogenous peroxidase was blocked for 30 min with fresh 3 % H2O2. Next, blocking with horse serum was carried out for 1.0–1.5 h with pre-washing in PBS. The duration and conditions of probing with primary antibodies were chosen according to the manufacturer’s instructions (sections with antibodies were incubated overnight at 4 °C). Next, after washing four times (PBS, pH 7.6), the tissue sections were incubated with secondary antibodies for 45 min, and then, after washing (PBS, pH 7.6), the tissue sections were incubated with diaminobenzidine as a substrate until a brown color appeared upon visual inspection. After that, the sections were counterstained with Mayer’s hematoxylin for 1 min, placed in tap water for 5 min, passed through a graded series of ethanol and xylenes, covered with the synthetic BioMount medium, and placed under a cover glass. Cells positive for staining with the above antibodies were counted in all subfields of view in the cortical and hippocampal areas highlighted with a black line in Figure 2, by a previously described approach (Pakharukova et al., 2019).

Statistics. To compare groups, two-way ANOVA and three-way ANOVA were performed in the STATISTICA 6.0 software, followed by a post hoc comparison of groups by the least significant difference (LSD) test. The following factors were analyzed during the intergroup comparison: “infection” (O. felineus or no O. felineus) and “ethanol” (ethanol or no ethanol). In the open field test, an additional within-group factor was “peppermint”: the first or second 3 min of observation, i. e., in the absence or presence of peppermint in the tumbler, respectively. The difference in the startle amplitude between the first and 10th stimulus in each group of mice was assessed by the Wilcoxon matched-pairs test. In all analyses, data with a p-value ≤ 0.05 were considered statistically significant, and those with 0.05 < p < 0.1 were regarded as an insignificant tendency. All data are given as means ± SEM.

## Results

Pathomorphological changes in the liver of mice of experimental groups

Control animals had normal liver architecture with well-defined portal triads (Fig. S1)1. On liver sections of animals from groups OF and Eth+OF, bile ducts appeared dilated, helminths were present in some of them, and there was a noticeable number of proliferating bile ducts and considerable lymphocytic–monocytic inflammatory infiltration. The bile duct epithelium became stratified in OF and Eth+OF mice. In the liver of animals from group Eth, fatty dystrophy of hepatocytes was noted.


Supplementary Materials are available in the online version of the paper: https://vavilovj-icg.ru/download/pict-2025-29/appx5.pdf


Masson staining of liver sections revealed expansion of connective tissue in OF and Eth+OF mice, both in the region of large bile ducts (periductal fibrosis) and in the region of proliferation of small bile ducts (cholangiofibrosis) (Fig. S2). It is noteworthy that the combination of the two adverse factors (infection and ethanol) featured a significantly aggravated course of opisthorchiasis, namely the severity of fibrosis and the size of infiltration loci; these signs were absent with each adverse factor applied alone. Additionally, in OF and Eth+OF animals, there was a change of epithelial cells consistent with intestinal metaplasia. Hemozoin granules were found in some mice, in agreement with what has been previously observed in O. felineus-infected Syrian hamsters (Lvova et al., 2016).

Histological analyses of the cerebral cortex

**Fig. 3.**
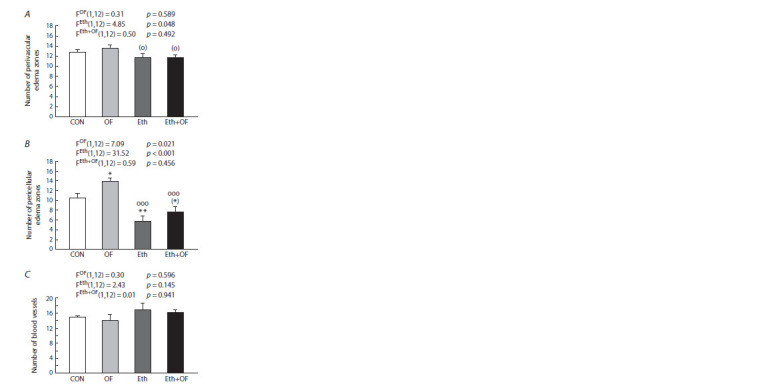
Changes in the number of perivascular (A) and pericellular edema foci (B) and blood vessels (C) in the cerebral cortex of control mice (CON), O. felineus-infected mice (OF), mice consuming 20 % ethanol (Eth), and mice subjected to both procedures (Eth+OF). * p < 0.05; ** p < 0.01; (*) 0.05 <p <0.1 as compared with group CON; ooo p < 0.001; (o) 0.05 < p < 0.1 as compared with OF mice. The values are presented as mean ± SEM and were processed by two-way ANOVA followed by the LSD test.

Histological examination of brain sections uncovered differences in the number of perivascular zones of edema (around the vessels) and pericellular zones of edema (around the cells) in the cerebral cortex. There was a somewhat greater number of perivascular edema zones in OF mice compared to Eth and Eth+OF mice (Fig. 3A); an effect of the ethanol factor on this parameter was detected. OF mice differed significantly from all other animals by having an increased number of pericellular edema zones (Fig. 3B); the effect of the infection factor was significant. Ethanol contributed to a decrease in this parameter, especially in mice from group Eth. Moreover, the ethanol factor had a >4 times more pronounced impact than the infection factor did; in the absence of their interaction, this outcome contributed to a lower value of this parameter in Eth+OF mice. The mouse groups did not differ in the number of vessels in the cerebral cortex (Fig. 3C). The assessed parameters in mice of all the experimental groups are presented in photographs of histological sections of the cerebral cortex (Fig. S3).

Immunohistochemical analysis of hippocampus and cortex sections

**Fig. 4.**
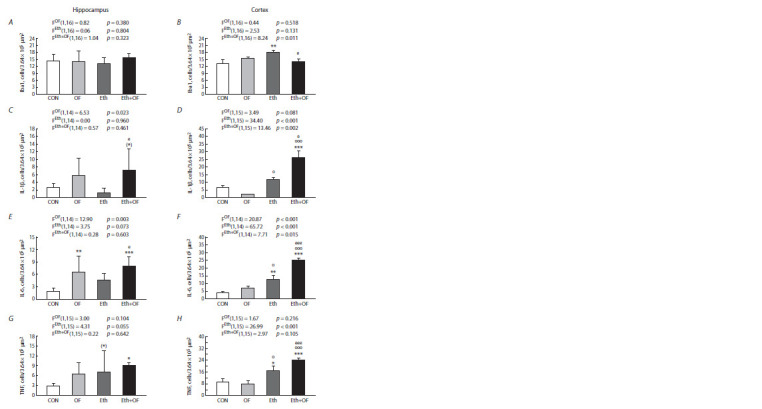
Numerical density of brain cells in the hippocampus (A, C, E, G) and cortex (B, D, F, H) stained for Iba1, IL-1β, IL-6, and TNF in the four mouse groups: control (CON), O. felineus-infected (OF), consuming 20 % ethanol (Eth), and subjected to both procedures (Eth+OF). * p <0.05; ** p <0.01; *** p <0.001; (*) 0.05 < p <0.1 in comparison with group CON; o p < 0.05 and ooo p < 0.001 in comparison with group OF; e p < 0.05 and eee p < 0.001 in comparison with group Eth. The values are presented as mean ± SEM and were processed by two-way ANOVA followed by the LSD test.

Analysis of Iba1-positive cells in the hippocampus revealed no difference among the groups (Fig. 4A). In the cerebral cortex (Fig. 4B), this parameter was higher in mice consuming ethanol (group Eth). Nonetheless, with a statistically significant interaction between the two adverse factors, this parameter decreased to the control level in Eth+OF mice. The number of IL-1β-positive cells was higher in both the hippocampus (Fig. 4C) and cortex (Fig. 4D) during combined treatments (Eth+OF). Although in the hippocampus, this phenomenon was determined by the influence of the infection factor, in the cortex, in addition to infection, this outcome was facilitated by an even more significant influence of ethanol, and there was a synergistic interaction of the two factors

A similar but even more pronounced pattern was documented for proinflammatory cytokine IL-6. In the hippocampus (Fig. 4E), infection to a greater extent and ethanol to a lesser extent contributed to a higher value of this parameter in Eth+OF mice. In the cortex (Fig. 4F), on the contrary, ethanol exerted a greater effect on an increase in the number of IL-6-positive cells, and a statistically significant interaction of the adverse factors caused a significantly higher value of this parameter in Eth+OF animals compared to the other three groups of mice (CON, OF, and Eth). There was a pronounced effect of ethanol, but not infection, on the increase in the number of TNF-positive cells in the hippocampus (Fig. 4G) and especially in the cortex (Fig. 4H). As in the analyses of the other proinflammatory cytokines, a statistically significantly higher value of this parameter was found in the cortex of Eth+OF mice relative to the other groups. Examples of immunohistochemical staining of the analyzed brain sections from the four mouse groups are presented in Figures S4 and S5.

Gene expression analysis in the hippocampus and cortex

**Fig. 5.**
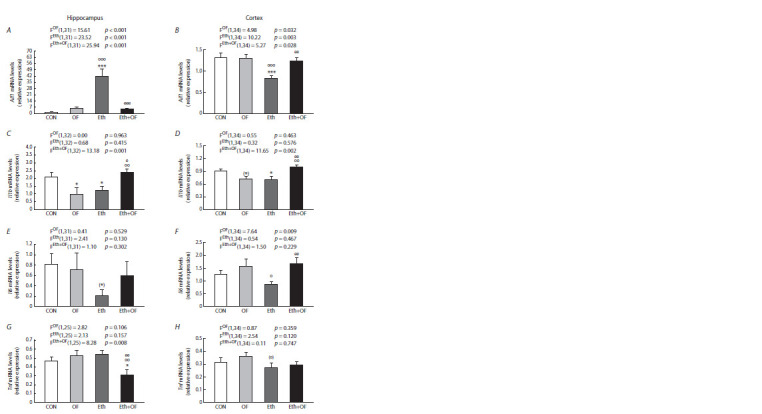
Relative levels of mRNA expression of genes Aif1, Il1b, Il6, and Tnf in the hippocampus (A, C, E, G) and frontal cortex (B, D, F, H) of mice from the four groups: control (CON), O. felineus-infected (OF), consuming 20 % ethanol (Eth), and subjected to both procedures (Eth+OF). * p <0.05, *** p < 0.001, (*) 0.05 < p <0.1 in comparison with group CON; o p < 0.05, oo p < 0.01, ooo p < 0.001, (o) 0.05 < p < 0.1 in comparison with group OF; e p <0.05, ee p < 0.01, eee p < 0.001 in comparison with group Eth.

Our quantitative analysis (qPCR) detected a significant effect of both adverse factors on the expression of the Aif1 gene in the two brain structures under study (Fig. 5A, B). In the hippocampus, ethanol consumption had a more pronounced effect, contributing to a 28-fold higher value of this parameter in Eth mice compared to CON mice and a 7–8-fold higher value compared to groups OF and Eth+OF. An interaction of the factors was noted to cause underexpression of the Aif1 gene in Eth+OF mice. In the frontal cortex, on the contrary, ethanol consumption contributed to Aif1 underexpression (the Eth group), and when the two adverse factors were combined, this parameter rose to the level of control mice.

When the expression of the Il1b gene was assessed in the hippocampus, no statistically significant effect of either factor alone was revealed, although a subsequent post hoc comparison of the groups detected a lower value of this parameter in mice from groups OF and Eth compared to CON mice. Nevertheless, a significant interaction of the two factors (Eth+OF mice; Fig. 5C) caused an increase in this parameter to the control level. A similar pattern was registered in the frontal cortex (Fig. 5D).

There was no statistically significant effect of each factor or of their interaction on the expression of the Il6 gene in the hippocampus (Fig. 5E). The infection factor had a significant effect on the Il6 expression in the frontal cortex (Fig. 5F), and this outcome contributed to an increase in the parameter in Eth+OF mice to the control level; this parameter was lower in Eth mice than in OF mice.

The combination of the two adverse factors caused downregulation of the Tnf gene in the hippocampus of Eth+OF mice compared with the other groups of animals (CON, OF, and Eth) (Fig. 5G). In the frontal cortex of the brain, the expression of this gene was approximately the same in all four groups of animals (Fig. 5H); no statistically significant influence of each factor or of their interaction was detectable.

The startle reflex

**Fig. 6.**
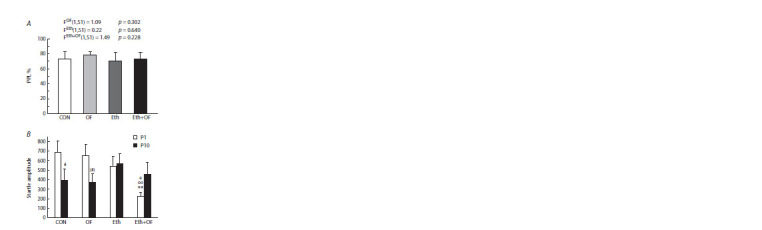
Prepulse inhibition (PPI) (A) of the startle response to an acoustic stimulus and startle amplitude (B) in response to the first (Р1) and 10th (Р10) stimulus in control mice (CON), O. felineus-infected mice (OF), mice consuming 20 % ethanol (Eth), and mice subjected to both procedures (Eth+OF). ** p <0.01 in comparison with group CON; oo p <0.01 in comparison with OF mice; e p <0.05 in comparison with group Eth; # p < 0.05 and (#) 0.05 < p < 0.1 in comparison with the Р1 data. The values are presented as mean ± SEM. Two-way ANOVA was used followed by the LSD test (A) and the Wilcoxon matched-pairs test (B).

Neither the prolonged consumption of ethanol nor chronic infection significantly affected PPI according to the evaluation of the startle response to acoustic stimuli (Fig. 6A). Nevertheless, the ethanol-drinking animals showed no habituation to the repetitive sound: in Eth mice, the startle response was the same for the 1st and 10th pulse with an intensity of 115 dB; the same was true for Eth+OF mice (Fig. 6B). Furthermore, in CON mice (statistically significantly) and in OF mice (insignificantly), the startle response to the sharp sound applied 10 times was found to decrease. It is noteworthy that in Eth+OF mice, the response to the first sound was the lowest when compared with the other groups, and there was a significant influence of ethanol [FEth(1,51) = 8.10, p = 0.006] and an insignificant influence of infection [FOF(1,51) = 3.09, p = 0.085], but there was no interaction of the two factors [FEth+OF(1,51) = 1.81, p = 0.185]. The reaction of the mice to the 10th sound signal was the same among the four groups.

The open field test

**Fig. 7.**
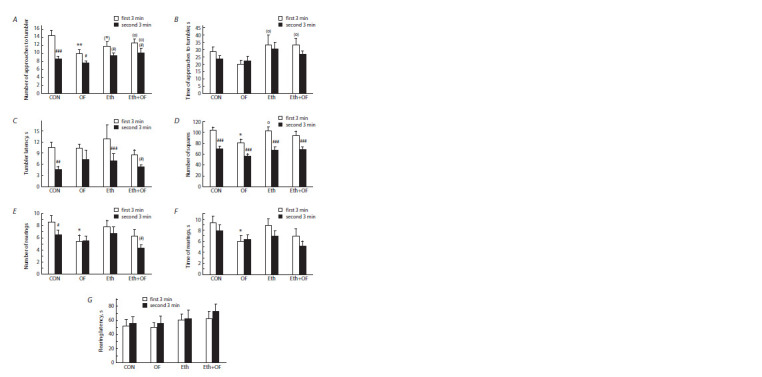
Behavioral parameters of control mice (CON), O. felineus-infected mice (OF), mice consuming 20 % ethanol (Eth), and mice subjected to both procedures (Eth+OF) in the open field test with an empty tumbler (the first 3 min) and a tumbler filled with peppermint leaves (the second 3 min). The number (A), time (duration) (B), and latency (C) of approaches to the tumbler, the number of square crossings (D), and the number (E), time (duration) (F), and latency (G) of rearings near a wall were determined. # p < 0.05, ## p < 0.01, ### p < 0.001, and (#) 0.05 < p < 0.1 as compared with the first 3 min of the test. * p < 0.05, ** p < 0.01, and (*) 0.05 < p < 0.1 as compared with the corresponding time slot in group CON. o p < 0.05 and (o) 0.05 < p < 0.1 as compared with the corresponding time slot in group OF. The values are presented as mean ± SEM. Three-way ANOVA followed by the LSD test.

In this test, intergroup differences were detected in the response of the mice to the odor of peppermint placed in the tumbler (Fig. 7A–C, Table S1). Even though the duration of staying near the tumbler with peppermint was approximately the same among all the groups, the number of approaches to it was lower in the second 3 min (i. e., when peppermint was present), especially in the CON group; the impact of the peppermint factor on this parameter was significant (Fig. 7A). An interaction of the ethanol and infection factors was detectable too (Table S1). Furthermore, CON and Eth mice sensed the peppermint smell faster than the other animals did because the latency period of the first approach to the tumbler with peppermint was significantly shorter in groups CON and Eth (Fig. 7C). In OF mice, this parameter did not differ from the latency period of approaching the empty tumbler, whereas in group Eth+OF, there was an insignificant decrease from the first 3 min to the second 3 min. This finding may reflect impairment of olfactory sensitivity in the course of prolonged parasitosis.

In the first 3 min of the test, the number of crossed squares (reflecting locomotor activity of the animals) was lower in the OF group than in groups CON and Eth (Fig. 7D); the effect of infection was statistically significant (Table S1). With a change in the motivation of mice after peppermint was placed in the tumbler, this parameter markedly diminished in all four groups of mice (Fig. 7D), and a significant impact of the peppermint factor was registered (Table S1).

Exploratory activity, assessed by means of the number and duration of rearings, was influenced by the infection and peppermint factors (Fig. 7E, F; Table S1). In the first 3 min of the test, these parameters were lower in OF mice than in CON mice. As compared to the first 3 min, the number of rearings in the second 3 min was significantly lower in CON mice and insignificantly so in group Eth+OF, whereas the duration of rearing stayed approximately the same in all groups. There was no influence of factors or of their interaction and no differences among the four groups in the latency of rearings either in the first or in the second 3 min of the test (Fig. 7G; Table S1).

The forced swimming test

**Fig. 8.**
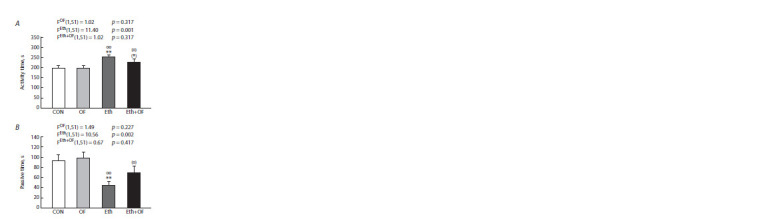
The duration of active (A) and passive swimming (B) in control mice (CON), O. felineus-infected mice (OF), mice consuming 20 % ethanol (Eth), and mice subjected to both procedures (Eth+OF) in the forced swimming test. ** p < 0.01 and (*) 0.05 < p < 0.1 as compared with group CON. oo p < 0.01 and (o) 0.05 < p < 0.1 as compared with group OF. The values are presented as mean ± SEM. Two-way ANOVA followed by the LSD test.

Prolonged consumption of ethanol had a significant effect on the behavior of mice in the forced swimming test. Activeswimming duration was longer (Fig. 8A), and passiveswimming duration was shorter (Fig. 8B), especially in mice of the Eth group and, to a lesser extent, in Eth+OF mice, both in comparison with CON and with OF mice.

## Discussion

This is the first study on the states of the liver and brain during exposure of mice to a combination of two adverse factors: infection (O. felineus helminths) and a toxic chemical (ethanol). Influence of each factor and of their combined action on the cortex and hippocampus was found in the brain of mice, as determined not only by molecular and histological methods but also at the level of behavior, which is considered a marker of functional changes in the brain

First of all, as previously (Avgustinovich et al., 2022a), it was shown here that both adverse factors, especially their combination, cause hepatic inflammation. In mice subjected to the combination of the factors, there was not only a bile duct pathology caused by the infection (cholangio- and periductal fibrosis and inflammatory infiltration) but also hepatocyte dystrophy induced by ethanol. These data – just as previously detected elevated blood levels of leukocytes (especially stab neutrophils and monocytes) and of IL-6 (Avgustinovich et al., 2022a) ‒ point to substantial inflammation in the body. It is known that virus- and alcohol-induced liver diseases are accompanied by inflammation and fibrosis associated with the suppression of activation of NF-κB in hepatocytes: a transcription factor that is a key regulator of inflammation and cell death in the liver (Seki, Schwabe, 2015). In this context, Kupffer cells increase their production of proinflammatory cytokines, including TNF, IL-1β, and IL-6 (Bilzer et al., 2006), with their subsequent traffic to the brain, where monocytes are also attracted from the bloodstream (D’Mello, Swain, 2011). As other researchers believe (Yang et al., 2014; Simon et al., 2019), these events are followed by activation of resident and recruited microglial cells, contributing to the development of neuroinflammation due to the production of inflammation mediators.

One of important indicators of inflammation in the brain is edema, the pathogenesis of which involves many factors, including liver failure (Adeva et al., 2012). In acute liver failure, edema is initially localized to the perivascular space and a zone of large swollen astrocytes. In our study, the experimental groups of mice did not differ from controls in perivascular edema, although in the animals consuming ethanol (groups Eth and Eth+OF), this parameter was somewhat less pronounced than that in OF mice. It is thought that when a pathological process in the liver is prolonged, structural changes occur in blood–brain barrier components (capillary endothelium, basal membrane, and astrocyte vascular peduncles), and these alterations reduce its protective function (Mishchenko et al., 1993). Blood–brain barrier destabilization facilitates penetration of “noxious agents” [e. g., ammonia, cytokines, or bacterial cell wall endotoxins: lipopolysaccharides (LPSs)] (Jayakumar et al., 2012) into the brain, with consequent pericellular edema due to the accumulation of fluid in the intracellular space, as demonstrated by magnetic resonance imaging in humans (Chavarria et al., 2011). We noticed significant predominance of pericellular edema in OF animals. At the same time, in the Eth+OF group of mice, ethanol “corrected” the number of edema foci, thereby reducing this parameter below the control value. Therefore, the actions of the two factors had opposite directions.

We assume that the increase in the number of pericellular edema zones during the helminth infection may proceed according to the hepatic-encephalopathy scenario, associated not only with upregulation of inflammatory cytokines (TNF, IL-1β, IL-6, and IFN-γ) in the blood but also with elevated blood and brain ammonia levels, as shown in acute and chronic liver failure (Butterworth, 2003; Rama Rao et al., 2014; Upadhyay, 2017). Ammonia is extremely toxic to the brain and leads to hepatic coma. Considering the evidence from other researchers that alcohol promotes edema in the brain (Collins et al., 1998; Collins, Neafsey, 2012; de la Monte, Kril, 2014), the decrease in the number of pericellular edema zones in mice consuming ethanol in our experiment remains unexplained. It can be hypothesized that aquaporins (AQPs) are involved, which are water-selective plasma membrane channels that enhance water permeability of cells (Huber et al., 2007), because there are reports confirming that ethanol can diminish swelling in the cortex after brain injury and that this phenomenon is associated with underexpression of AQP4 and AQP9 simultaneously with an improvement of cognitive and motor functions in animals (Wang et al., 2013). Because it is known that aquaporins are enriched within brain astroglia (Huber et al., 2007; Collins, Neafsey, 2012), the decrease in pericellular edema during prolonged ethanol consumption in our experiment may be related to its degenerative effect on astrocytes (de la Monte, Kril, 2014). As demonstrated by the optical fractionation technique, in severely ill alcoholics, there is a loss of 37 % of glial cells in the hippocampus, primarily astrocytes and oligodendrocytes and to a lesser extent microglial cells, without a loss of neurons (Korbo, 1999). Apparently, our data require further research.

It is known that brain microglia, when responding to any pathological stimulus coming from the periphery (an LPS challenge, vaccination, or alcohol), begin to produce proinflammatory cytokines with subsequent stimulation of a release of cytokines and chemokines from neurons and astrocytes (Miller et al., 2009; Yang et al., 2014; Norden et al., 2016; Henriques et al., 2018).

According to our findings, there are brain region-specific changes in the expression of the Aif1 gene, reflecting the activity of microglia, during the exposure to the two adverse factors. In the hippocampus, where microglial density is reported to be the highest (Silvin, Ginhoux, 2018; Tan et al., 2020), a statistically significant influence of both factors and of their interaction was documented in our work. Chronic consumption of 20 % ethanol contributed to a significant increase in this parameter; the impact of the infectious factor was less pronounced but statistically significant, as found previously (Avgustinovich et al., 2022а). It is important that when the two factors acted together, Aif1 expression in the hippocampus was low. Apparently, in this situation, activation of microglia cells in the hippocampus is so substantial that the activated microglia can be driven into apoptosis, in order to prevent the brain from entering a state of chronic inflammation. Currently, the mechanisms controlling microglial apoptosis are characterized incompletely, but the possibility of a chain of events (in the brain) proceeding from the stage of activated microglia to their apoptosis has been examined by other researchers (Fu et al., 2015). Further microscopic research on phenotypic alterations in microglial cells is necessary to identify their transformation from a resting state (ramified morphology) to active status (amoeboid morphology) (Tan et al., 2020).

In the frontal cortex, a significant influence of each adverse factor and of their statistically significant interaction on the expression of the Aif1 gene was noted too. In contrast to the hippocampus, in this brain structure, ethanol reduced this parameter, and the combination of the two factors contributed to its increase to the level of control individuals. Evidently, the infectious factor has an effect opposite to that of ethanol, thus increasing the expression of this gene. Immunohistochemistry revealed a statistically significantly greater number of Iba1- positive cells in the cortex in a more distant frontal section (level 74) as compared to the prefrontal region of the brain. Therefore, we can theorize that the observed downregulation of Aif1 in Eth mice is a compensatory reaction of genes to the large protein amount.

Thus, in our paper, region-specific changes in microglial activity were established in terms of the expression of the Aif1 gene and of a protein (Iba1) in response to the infectious factor (O. felineus), and especially to the toxic factor (ethanol). This result can be explained in accordance with the current understanding of the heterogeneity of microglia in the cell number, morphomolecular signatures, and homeostatic functions in different anatomical structures of the healthy CNS (Tan et al., 2020) and during alcoholic pathologies (He, Crews, 2008). In this regard, our data indicate a possible modifying effect of helminth infection on the expression of the Aif1 gene, but not its protein, in the hippocampus and cortex. Ethanol had a more pronounced effect on this parameter in both brain structures. Taking into account the interaction of the two factors, we can say that their effects have different directions. Activation of microglia in the cortex under the influence of ethanol was also recorded in terms of the level of the Iba1 protein

It has been reported that peripheral insults (an LPS or bacterial challenge or ethanol) that cause microglial activation induce upregulation of proinflammatory cytokines (TNF, IL-1β, and IL-6) at the protein and/or mRNA level along with engagement of Toll-like receptors (TLR-2 and TLR-4) (Collins, Neafsey, 2012; Fernandez-Lizarbe et al., 2013; Yang et al., 2014; Hoogland et al., 2015). As reported by C. D’Mello and M.G. Swain (2011), cytokines TNF, IL-1β, and IL-6 are likely to be key promoters of central neural alterations in chronic liver diseases. Accordingly, we next examined changes in expression of three proinflammatory cytokines (IL-1β, IL-6, and TNF) in response to the adverse factors.

The expression of the Il1b gene in the two brain structures under study did not reflect a statistically significant effect of each factor alone, but there was a significant effect when they were combined: the weak expression of this gene in groups OF and Eth increased in Eth+OF mice to the control level. Immunohistochemical analysis of sections of the hippocampus, and especially that of the cerebral cortex, pointed to high levels of this proinflammatory cytokine in mice during the exposure to the two factors, thereby implying neuroinflammation

We believe that our results on protein and mRNA levels of Il6 in the hippocampus reflect a variable process associated with the duration of the adverse factors: a change in the amount of the proinflammatory protein was followed by a change in mRNA expression, and these processes seem to be somewhat separated in time. In the cerebral cortex of Eth+OF mice, there was the highest level of IL-6 among all groups of mice, which is explained by the synergy of the two adverse factors. Nonetheless, there were no pronounced differences between the groups in Il6 expression within this brain structure, and this outcome may be ascribed to regulation by a negative feedback mechanism. Similar dynamics of expression (from unchanged to increased/decreased) of genes responsible for levels of IL-6, IL-1β, and TNF in the three structures of the rat brain were recorded by other researchers after forced consumption of ethanol by rats (for 6 months) (Nunes et al., 2019). Those authors proposed that the upregulation of some cytokines may mean infiltration of immune cells (T cells in particular) into the brain, and these phenomena are indicative of a severe impairment of the blood–brain barrier especially during the synergism of the two adverse factors. Under such conditions, the delayed changes in the genes’ expression may have a protective/compensatory effect against expansion of neuroinflammatory-cytokine expression in the brain.

In contrast to the other two genes, the combination of the helminth infection and the prolonged ethanol consumption caused underexpression of Tnf in the hippocampus of Eth+OF mice; this phenomenon may be a compensatory reaction to the elevated amount of the TNF protein in this brain structure. An even higher level of this protein was detected in the cortex at level 74 of the frontal brain section during ethanol administration, but the Tnf mRNA level in the frontal cortex was the same among all the groups of mice.

Thus, three proinflammatory cytokines were found to differ in mRNA or protein expression depending not only on the nature of the adverse factor(s) but also on localization in the brain. The most pronounced variations of the parameters were noticed in the frontal cortex, especially during prolonged ethanol consumption, and to a lesser extent in the hippocampus. It is known that both brain structures in question, especially frontal lobes of the cerebral cortex, are sensitive to alcohol-induced damage (Fowler et al., 2014). In this regard, the pathogenic effect of alcohol is associated with white matter atrophy, neuroinflammation, and synaptogenesis disturbances, leading to emotional instability and cognitive impairment (Harper, 2009; de la Monte, Kril, 2014). The outcomes observed here – just as previously obtained evidence that inflammation in the liver during the action of the two adverse factors is accompanied by an increase in blood concentrations of monocytes and proinflammatory cytokines (Avgustinovich et al., 2022a) – are thought to contribute to neuroinflammation and may induce changes in central neurotransmission that are manifested in the behavior of animals (D’Mello, Swain, 2011). That is why we performed an extensive analysis of murine behavior, which reflects disturbances in the brain.

Even though PPI was the same among our groups of mice, the animals in the groups consuming 20 % ethanol for a long time (Eth and Eth+OF) did not get accustomed to the administered signals: startle amplitudes of the 1st and 10th signals were the same. In addition, the mice subjected to both adverse factors (Eth+OF) showed the weakest reaction to the first sharp sound signal, but the response increased by the 10th signal. These results mean that the brain of mice consuming ethanol is always ready to respond strongly to repeated harsh sound signals. Considering that the startle reflex is regarded as a behavioral indicator of CNS excitability (Blendov et al., 2019), we can assume permanent high excitability of brain neurons in mice consuming 20 % ethanol

Throughout almost the whole forced swimming test (on average 252 out of 300 s), mice of the Eth group tried to actively get out of the water, and passive swimming was shorter. In Eth+OF mice, the changes in these parameters were smaller: the differences from groups CON and OF were insignificant. The duration of immobility in this test is regarded as an indicator of depressive-like behavior in rodents and is reduced by known antidepressants (Lucki, 2001). Nonetheless, we believe that the prolonged consumption of 20 % ethanol did not have an antidepressant effect but rather promoted CNS hyperexcitability, which involves an imbalance in the activities of the glutamatergic and GABAergic systems of the brain. It is known that chronic alcohol consumption leads to hyperexcitation of neurons because of downregulation of GABAergic functions as a consequence of pseudo-immaturity in the hippocampus and prefrontal cortex (Murano et al., 2017). А blockade of NMDA receptors and of the nitric oxide/cyclic-guanosine monophosphate pathway may be involved in the antidepressant-like effect of ethanol in mice (Khan et al., 2021).

The open field test in its various modifications is utilized by researchers to assess many behavioral parameters in rodents: locomotor and exploratory activities, emotionality and anxiety, and a reaction to an unfamiliar object when re-tested (Choleris et al., 2001). Because our aim was, first of all, to assess the sense of smell in the four groups of mice, the unfamiliar smell of peppermint was presented to the mice during the second part of the test, after they were familiarized with the test arena in the first 3 min. New odors are often aversive to rodents, for example, rats avoid the peppermint smell at first exposure (Brown, Willner, 1983). On the other hand, in some studies on mice, investigators have described repeated use of peppermint for treating olfactory impairment (Kim et al., 2019).

In our experiments, mice subjected to prolonged ethanol consumption (group Eth), just as CON mice, quickly identified the unfamiliar odor because their latency period for approaching the tumbler with peppermint diminished. The infectious factor did not affect this parameter in OF mice and had a weak effect on Eth+OF mice, implying disturbances of the sense of smell in these mice. Besides, although the control mice exhibited a pronounced avoidance reaction toward the tumbler with peppermint (as evidenced by the number of approaches or turns to the tumbler), in the other groups of mice, this parameter was less pronounced. This result also points to some anomalies in the sense of smell in mice of the three experimental groups [subjected to an adverse factor(s)]. We believe that the changes in the sense of smell resulting from liver fluke infection and consumption of 20 % ethanol may be associated with aberrations in the CNS. In any case, hyposmia is considered an early symptom of Parkinson’s disease (Chen et al., 2012), which is also associated with neuroinflammation.

During the evaluation of other patterns of behavior in the open field test, it was found here that the 6-month consumption of 20 % ethanol did not have a significant effect on the locomotor and exploratory activities of mice, as evaluated via the number of squares crossed and rearing parameters in the first 3 min of the test. By contrast, these parameters were lower in OF mice. A decrease in these parameters, according to other articles (Henderson et al., 2004; Seibenhener, Wooten, 2015), can be viewed as a manifestation of anxious behavior. Taking this into account, we could say that there is a likely proanxiety effect of prolonged O. felineus infection on animals.

## Conclusion

The presented data are an experimental model of situations often occurring in human society: people with chronic opisthorchiasis – perhaps being unaware of the infection – abuse alcohol or, conversely, by relying on disinfecting properties of ethanol, begin to drink it, sometimes in large amounts, when there is a threat of this infection. Nonetheless, our experimental data indicate that under such circumstances, the liver is not the only organ that receives a double “blow” (the toxic injury plus the infectious one). When chronic alcoholization is combined with prolonged O. felineus infection, the brain also receives a double impact: aside from direct entry of ethanol into the CNS through the blood–brain barrier, according to D’Mello and Swain (2011), peripheral proinflammatory signals begin to arrive with the blood, primarily IL-1β, IL-6, TNF, and monocytes.

Under these conditions, as revealed by two-way ANOVA, there are statistically significant effects of interaction of the two adverse factors on histological and molecular characteristics of microglia and on proinflammatory cytokines, and these effects are brain region-specific. For instance, in the hippocampus, the infectious factor attenuated ethanol-induced Aif1 overexpression, which reflects the activation of microglia. By contrast, in the frontal cortex, the expression of this gene was low during the prolonged alcoholization and increased to control values in the mice subjected to both factors. During a statistically significant interaction of the factors, this finding indicates that directions of the two impacts are different. It is possible that helminths exert a “corrective” effect here that is designed to preserve the health of the host (at whose expense they live and reproduce) because excessive activation of microglia can have irreversible neurodegenerative consequences and may ultimately kill the host. Identical directions of the effects of the two factors were noted during quantification of the expression of the Il1b gene (in the cortex and hippocampus) and of the Tnf gene (in the hippocampus): these effects promoted an increase in the former parameter and a decrease in the latter and may be attributed to the proteins’ levels at this stage of the pathology

In the cortex, high concentrations of cytokines IL-1β, IL- 6, and TNF were found at levels 72–74 of the frontal brain slices in mice subjected to both factors; this finding implies that the alterations induced in the prefrontal region of the cerebral cortex might be similar. Together with an increase in these parameters in the hippocampus, this finding indicates the development of neuroinflammation.

We believe that the obtained results indicate a variable process that is largely explained by the duration of the stimuli: a change in the amounts of proinflammatory proteins is followed by a change in mRNA expression. Furthermore, these processes are brain region-specific and seem to be somewhat separated temporally. This is because these processes not only are regulated by the activity of brain cells but also depend on the arrival of peripheral proinflammatory signals into the brain. This phenomenon in turn affects the behavior of the animals. Behavioral testing of our mice revealed that ethanol has a stimulatory effect, which manifested itself in two tests reflecting alterations in the regulatory mechanisms of the CNS. In our mice, the behavioral pathology associated with O. felineus infection is suggestive of the development of anxiety. It should be pointed out that both factors altered the mice’s sense of smell (the infection did so to a greater extent). Mechanisms that counteract these adverse effects remain to be explored. At this stage of the project, we can state only that the combination of the two factors – in addition to the destructive effects on the liver – can affect the brain too, by contributing to the development of neuroinflammation.

## Conflict of interest

The authors declare no conflict of interest.
